# Tartar on Teeth

**Published:** 1852-07

**Authors:** 


### ARTICLE VII.

### *Tartar on Teeth.*

THE salivary calculus or earthy concretion found upon the teeth, is called tartar; and, may be regarded as a type of the fluid from which it has been deposited. With the exception of caries, nothing is so destructive to the healthy condition of the mouth, and the duration of the teeth, as tartar; nor more offensive to the sight; while it cannot but prove equally injurious to health, as other bad smells. Tartar inflames and separates the gums from the teeth; and inflamed gums produce absorption of the bony sockets, in which the teeth are fixed; when, as a matter of course, they loosen and drop out. While the salts and other materials, of which tartar is composed, are yet in a soft, viscous, and adhesive state—their natural condition when first precipitated from the saliva, of which they form an important part, the deposition is called fur; and fur, as most people are aware of, is deposited on the tongue, and other parts of the mouth, as well as on the teeth. In this state, however, it is necessarily wiped away by the action of the tongue, lips, and food during mastication, from every part naturally subject to friction; and, obviously, admits of easy removal from the less accessible localities, by the judicious use of tooth-brushes, tooth-picks, and successful rinsing with water, or other approved washes. And we constantly find, that the prominent parts of teeth, like those parts of machinery in motion, which come in contact with others, are in a high state of polish, cleanliness and health; while the lines and seeming cracks, natural to their surfaces are foul, and disposed to decay; and while their interstices (which unfortunately go on increasing in size as the gums recede) are filled up with fur; which, in due time, hardens to tartar; and tartar itself, becomes constantly harder the longer it remains.

A first year's deposit, for example, is more easily removed than that of a second, on account of the great hardness of the latter. There is a certain line, a sort of tidal beach running horizontally along both rows of teeth, above which, tartar is not permitted to collect, owing to the action of food during mastication; but, there is no limit to its deposition underneath, where the gums and teeth intersect each other; and, it accordingly eats away the entire substance of the sockets from sound teeth, substituting its horrid self to their very tips; and adhering with a tenacity which has caused it to be sometimes mistaken for bone, and more strangely still, it has been absurdly supposed to be a provision of nature, to support the teeth after the loss of their natural sockets. The materials of tartar at any stage retain liquids like chalk; and stagnant saliva, under the decomposing influence of the warm, moist atmosphere of the mouth, soon becomes rotten, and occasions offensive breath, alike unpleasant and unhealthy, and old dark tartar, buried under spongy, scorbutic gums, and the habitually retained relics of decaying food, soften the bone; which softening is in reality the natural history of caries in teeth; and foul tartar is said not unfrequently to teem with microscopic life. Tartar consists principally of carbonate of lime, animal, and other matters, the relative proportions of which, however, differ so much in different persons, and in the same persons, at different times, that any two analyses rarely furnish the same results, as may be seen from the three following accurate analyses furnished to the Lancet, by Dr. Wright:

| | 1st. | 2nd. | 3rd. |
|---|---|---|---|
| Carbonate of lime, . . . . | 81.3 | 79.4 | 80.7 |
| Phosphate of lime, . . . | 4.1 | 5.0 | 4.2 |
| Muriates, hydro-sulphocyanates, lactitis and carbonates, . . . (potash and soda.) | 6.2 | 4.8 | 5.1 |
| Animal matter, consisting of ptyaline, albumen and mucus, . . . | 7.1 | 9.5 | 8.3 |
| Loss in experiments, . . . . | 1.3 | 1.3 | 1.7 |
| | 100.0 | 100.0 | 100.0 |

There are, comparatively, few persons in civilized life, whose teeth remain permanently free from tartar; and many, from constitutional causes, that appear to be harmless in other respects, are much more subject to it than others. Even in animals, when confined in menageries or pampered as pets, and having no longer the same occasion or opportunity of exercising them as formerly; the teeth become rusty, and loaded with tartar; and with the same results, as in the analogous case of human beings. In accounting for the existence of tartar, it may be observed, that the animal fluids, when out of the course of general circulation, are apt to stagnate, and deposit sedimentary matter.

That, although during health, the flow of saliva is sufficiently copious to carry off in the food its more solid contents, as swollen streams scour their beds, yet, on the other hand, when vitiated by disease, its supply becomes scanty, sluggish and viscous; and forms an inspissated mucus, not on the teeth alone as already observed, but on the tongue and other parts of the mouth. With a barnacle-like grip, it fixes on the more rigid surfaces of the teeth, and remains on those parts only that happen to lie beyond the reach of friction during mastication, till it becomes tartar. Fur settles down in their layers, like tidal deposits upon the teeth and gums, and in their interstices, filling up and leveling with its pernicious substance, the breaches itself had made; and in such a manner as often to produce the deceitful appearance of a beautiful and perfect mouth, which it would seem a pity to break up; although, like a snake in the grass, it is all the while actively employed in destroying every thing within its reach.

In the absence of tartar, and with sufficient employment in masticating plain food, or wanting that, with successful brushing—the gums ought to be, like other healthy skin, thin, free from bleeding, and in apparent adhesion to the teeth; while below healthy gums, the sockets of teeth ought never to become absorbed, nor in any way diminished; nor do teeth loosen and drop out until the sockets have first been destroyed. There are two methods by which tartar may be kept off the teeth; the one natural—as when the teeth are well employed, as by barbarians, an abundance of plain food, the relics of which do not remain about them at any time; the other, artificial—as when teeth are successfully brushed, picked, and rinsed, throughout their interstices every day. The teeth of dogs and cats, running at large, are, in general, beautifully clean; and may be taken as examples of natural cleaning; while those of pets of either kind, are subject to the same diseases as those which attack human beings. J. P. C.

